# Deep Recurrent Neural Networks for Automatic Detection of Sleep Apnea from Single Channel Respiration Signals

**DOI:** 10.3390/s20185037

**Published:** 2020-09-04

**Authors:** Hisham ElMoaqet, Mohammad Eid, Martin Glos, Mutaz Ryalat, Thomas Penzel

**Affiliations:** 1 Department of Mechatronics Engineering, German Jordanian University, Amman 11180, Jordan; mutaz.ryalat@gju.edu.jo; 2 Department of Biomedical Engineering, German Jordanian University, Amman 11180, Jordan; m.eid1@gju.edu.jo; 3 Interdisciplinary Center of Sleep Medicine, Charité-Universitätsmedizin Berlin, 10117 Berlin, Germany; martin.glos@charite.de (M.G.); thomas.penzel@charite.de (T.P.)

**Keywords:** sleep apnea, deep learning, recurrent neural network, long short-term memory, sleep-disordered breathing

## Abstract

Sleep apnea is a common sleep disorder that causes repeated breathing interruption during sleep. The performance of automated apnea detection methods based on respiratory signals depend on the signals considered and feature extraction methods. Moreover, feature engineering techniques are highly dependent on the experts’ experience and their prior knowledge about different physiological signals and conditions of the subjects. To overcome these problems, a novel deep recurrent neural network (RNN) framework is developed for automated feature extraction and detection of apnea events from single respiratory channel inputs. Long short-term memory (LSTM) and bidirectional long short-term memory (BiLSTM) are investigated to develop the proposed deep RNN model. The proposed framework is evaluated over three respiration signals: Oronasal thermal airflow (FlowTh), nasal pressure (NPRE), and abdominal respiratory inductance plethysmography (ABD). To demonstrate our results, we use polysomnography (PSG) data of 17 patients with obstructive, central, and mixed apnea events. Our results indicate the effectiveness of the proposed framework in automatic extraction for temporal features and automated detection of apneic events over the different respiratory signals considered in this study. Using a deep BiLSTM-based detection model, the NPRE signal achieved the highest overall detection results with true positive rate (sensitivity) = 90.3%, true negative rate (specificity) = 83.7%, and area under receiver operator characteristic curve = 92.4%. The present results contribute a new deep learning approach for automated detection of sleep apnea events from single channel respiration signals that can potentially serve as a helpful and alternative tool for the traditional PSG method.

## 1. Introduction

The American Academy of Sleep Medicine (AASM) defines sleep apnea as the most common sleep-related breathing disorder [1]. Sleep apnea is characterized as a transient or complete cessation of breathing during sleep [1,2]. If breathing is only reduced, then the respiratory event is called a hypopnea. Sleep apnea can be classified into three major categories: Obstructive, central, and mixed apnea [3]. Obstructive sleep apnea (OSA) occurs when cessations in breathing during sleep are caused by the obstruction or collapse of the upper airway. Central sleep apnea (CSA) involves a neurological sleep condition which causes the loss of all respiratory effort while the airway is not necessarily obstructed. Mixed sleep apnea (MSA) combines both CSA and OSA, where a failure in breathing effort is followed by a collapse of the upper airway.

Obstructive sleep apnea (OSA) is the most common type among the general population. Undiagnosed OSA is a risk factor for very dangerous complications such as coronary artery disease, hypertension, cardiac arrhythmias, stroke, and diabetes [4,5]. OSA occurrence among adult men (24%) is higher than adult women (9%) [6]. There are over 200 million OSA patients all over the world [7].

A nocturnal polysomnography (PSG) is a standard multi-parametric test to diagnose and detect sleep breathing disorders [1,8]. However, PSG requires uncomfortable diagnostic equipment with multiple sensors, trained attendees, and great experience. Standard PSG signals include electrocardiogram (ECG), electroencephalogram (EEG), electromyogram (EMG), oxygen saturation of blood (SpO${}_{2}$), oronasal thermal airflow signal (FlowTh), and nasal pressure signal (NPRE) [1,8]. Additionally, the manual annotation process by sleep specialists is time consuming and labor-intensive. Different results can be produced and human errors can occur due to intraobserver and interobserver variability when performing manual scoring [9].

Over the last two decades, there have been several studies of novel apnea detection methods based on the study of a limited set of signals among those involved in PSG [10]. Thus, ECG, SpO${}_{2}$, and various respiratory signals have been utilized to help in sleep apnea diagnosis [3,11,12,13,14,15,16,17]. These studies followed a common methodology: Extract discriminative features, select optimal features, and apply them to different machine learning algorithms. However, those studies had some drawbacks because of numerous calculations and computations, handcrafted feature sets, and lower detection rates.

Recently, deep learning methods have been proposed and used for apnea detection to overcome the problems associated with manually extracted features and to improve the detection rates. A lot of studies used deep learning in the form of convolutional neural networks (CNN), which have shown high performances [18,19]. Nevertheless, CNNs are fundamentally designed for image recognition and normally require high computational power [20].

Recurrent neural networks (RNN) are extensions of classical feedforward neural networks. They have been shown to handle efficiently variable-length sequences and time-series data [21]. They have shown excellent performance in speech recognition and natural language processing applications [22,23,24]. In particular, the repetitive temporal occurrence of sleep breathing disorders can potentially make RNNs more useful and appropriate than conventional machine learning and/or CNN-based methods.

The contribution of this paper is two fold. First, we propose a novel method for automatic detection of apneic events based on deep RNN using only a single channel respiration signal. Second, we evaluate the performance of the proposed approach on 3 different respiration signals. We perform a comprehensive comparison between the performance achieved over each of the signals considered using different RNN detection scenarios.

This paper is organized as follows. Section 2 summarizes background and previous studies for detecting sleep apnea using PSG respiration signals. Section 3 describes the data set, the details of the proposed algorithm, and the evaluation metrics used in this study. Section 4 discusses results for the proposed algorithm which are further analyzed and investigated in Section 5. Finally, Section 6 summarizes the conclusion of this paper.

## 2. Background & Problem Statement

### 2.1. Standards for Scoring Sleep Apnea

The American Academy of Sleep Medicine (AASM) specifies the use of two respiration signal channels in order to detect respiratory events during PSG diagnostic studies. The first one is obtained through an oronasal airflow sensor and the second one is obtained through a nasal pressure sensor [25,26]. The oronasal airflow sensor is a thermal-based sensor in which its measuring principle is based on detecting the change in temperature between inhaled and exhaled gas. The technology used in the oronasal airflow sensor includes thermistors, thermocouples, or polyvinylidene fluoride (PVDF) sensors [27]. The nasal pressure sensor is composed of a nasal cannula connected to a pressure transducer. Unlike oronasal thermal airflow sensors that can detect both nasal and oral airflow, nasal pressure sensors can not detect oral airflow [28,29].

The thoracoabdominal movement sensor is recognized by AASM as the primary sensor for detecting respiratory effort as well as an alternative sensor for detection of sleep apnea and hypopnea. Thoracoabdominal movements record changes in the volume of the chest and abdomen over the breathing cycle providing an indirect air flow measurement from which a reduction of amplitude and alteration of the inspiratory flow curve can be detected [30]. The technology available for respiratory effort belts includes strain gauges, impedance plethysmography, respiratory inductance plethysmography, and belts with piezoelectric or PVDF sensors [27,31,32,33].

### 2.2. Algorithms for Automated Apnea Detection with PSG Respiration Signals

Due to their convenience, several studies have focused on automated detection of sleep apnea events based exclusively on the analysis of PSG respiration signals. Oronasal airflow, nasal pressure, and respiratory inductance plethysmography (RIP) signals have been used to extract features. Those signals were typically analyzed and investigated using advanced signal processing techniques in different analytic domains (time, frequency, linear, and nonlinear domains). Then, robust classifiers were used to discriminate between the classes of apnea and non-apnea segments. Examples of classification algorithms that have been used with respiratory signals include threshold-based detectors [34,35,36,37,38], support vector machines (SVM) [39,40], artificial neural networks (ANN) [41,42,43,44], as well as linear discriminant analysis (LDA) combined with regression trees (CART) and the boosting algorithm AdaBoost (AB) [45].

Recent studies that investigated deep learning methods in sleep apnea show improvement over classical machine learning methods [46]. While the majority of studies considered ECG signal [18,19,20,47,48,49,50,51,52,53], a very limited number of them investigated respiration signals. Research efforts with respiratory signals commonly considered multi-channel signal inputs for deep learning. This includes the oronasal/nasal airflow together with abdomen and thoracic plethysmography [54] including SpO${}_{2}$ [55,56] or ECG [57]. Very few studies considered single channel respiration signals [58,59,60,61]. Although single channel respiratory methods showed promising preliminary results, they only employed CNNs and did not evaluate recurrent neural networks. They also did not compare performance across different respiration signal inputs.

### 2.3. Problem Statement

Unlike previous studies that commonly leverage deep learning methods for automated detection of sleep apnea using ECG and/or other multi-channel signal inputs, this paper presents a deep learning framework for automated sleep apnea detection using single channel respiratory signals. Recognizing existing computational limitations of current CNN-based methods, we propose a deep recurrent neural network approach for automatic extraction for temporal features and automated detection for apnea events over successive 10-second windows in single channel respiratory signals. The proposed framework overcomes classical machine learning methods for apnea detection, which typically depend on the respiratory signal analyzed and feature extraction methods. Two major RNN models are used: long short-term memory and bidirectional long short-term memory. The proposed framework is evaluated over three respiration signals: Oronasal thermal airflow (FlowTh), nasal pressure (NPRE), and abdominal respiratory inductance plethysmography (ABD). A comprehensive comparison is demonstrated between apnea detection performance across the different signals using two different deep RNN detection scenarios: An LSTM-based model and a BiLSTM-based model.

## 3. Materials and Methods

### 3.1. Data Set

For this study, we used polysomnography (PSG) data for 17 patients recorded at the Interdisciplinary Center of Sleep Medicine in Charité- Universitätsmedizin Berlin in Berlin, Germany. PSG consisted of electro-oculography (EOG), electrocardiography (ECG), electroencephalography (EEG), submental and tibial electromyography (EMG), two belts for recording plethysmography respiratory inductance plethysmography (RIP) signals for thoracic (THO) and abdominal (ABD) wall motions respectively, an oronasal airflow sensor (FlowTh), nasal air pressure transducer (NPRE), pulse oximeter (SpO${}_{2}$), and a digital microphone.

Sleep apnea events in the data set were annotated and scored by expert clinicians from the Interdisciplinary Center of Sleep Medicine in Charité- Universitätsmedizin Berlin (Berlin, Germany). Scoring was carried out according to recommendations of the American Academy of Sleep Medicine (AASM) [1]. Apneic events in the data set are either obstructive (OSA), central (CSA), or mixed (MSA) ones.

### 3.2. Data Preprocessing

The airflow (FlowTh) and the RIP abdominal (ABD) signals were sampled at 32 Hz. The nasal pressure NPRE signal was sampled at 256 Hz. All of these signals were filtered with a low pass finite impulse response (FIR) filter with cutoff 0.5 Hz for preprocessing. The NPRE signal was down-sampled to 32 Hz so that all respiration signals had the same sample rate. Then, all preprocessed respiration signals were segmented at 10-s duration events. The segmentation was performed with no overlap. If more than half of a segment is annotated as normal, it was considered a normal event, and vice versa. The apneic segments were either obstructive, central, or mixed apnea events. The distribution of the data set with segments and corresponding labels is shown in Table 1.

As can be seen in Table 1, the data set was divided randomly such that 80% of the segments are used for training the deep learning networks with different sources of respiration signals while the other 20% of the segments are then used for evaluating the performance of these models in detecting apneic events. The same distribution of segments was used for each of the respiration signals considered in this study.

Table 1 also demonstrates that there is a clear class imbalance where the ratio of normal events to apnea events is nearly 4:1. Class imbalance is typical in sleep apnea problems and was overcome by oversampling the minority class (the apnea class) in the training data set.

Finally, to validate the proposed deep learning framework on a patient level, we employed a leave one out (LOO) approach. In this approach, we held out one patient data file each time and used PSG data from the remaining patients to build the deep learning model which was then evaluated on the held out patient data. This process was repeated on all patients iteratively until testing all patients in the data set.

### 3.3. Recurrent Neural Network (RNN)

Recurrent neural network (RNN) is a type of neural networks that is usually applied to the signal which has a correlation between its values during the time. Whereas common neural networks consider all values of the input signal that are independent. Fundamentally, an RNN is a looped-back architecture of interconnected neurons and current input whereby the last hidden state affects the output of the next hidden state. An RNN is ideally suited to sequential information and is excellent for time-series data because it also has memory [20].

The main advantage of the RNN is considering temporal dependencies and extracting temporal features. RNNs can add a loop of information flow. This means that previous units could alter and aid in the next instant of the process. During training RNNs with backpropagation through time (BPTT) [62], when the gradients are propagated over time, they tend to vanish or explode (become unstable) [63]. This problem makes it very difficult for RNNs to learn long time dependencies. To address this shortcoming, variations of RNN, such as long short-term memory (LSTM) and bidirectional LSTM (BiLSTM) can be used. LSTM/BiLSTM addresses the aforementioned problem and can also capture richer contextual information within sequences and time series.

#### 3.3.1. Long Short-Term Memory (LSTM)

**Figure 1:**
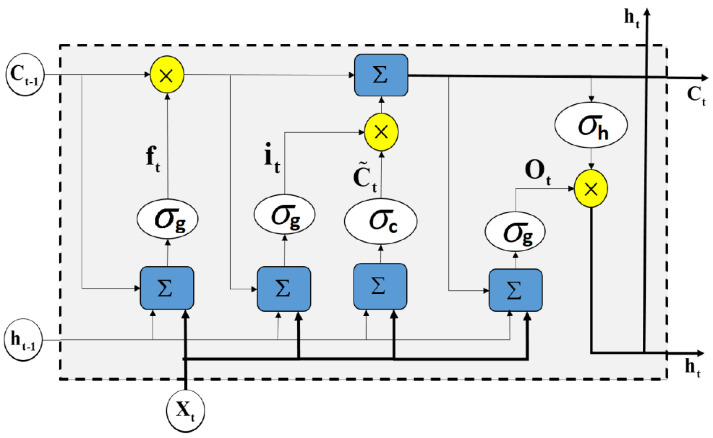
A typical architecture of a long short-term memory (LSTM) cell. An LSTM block typically has a memory cell, input gate ($i_{t}$), output gate ($O_{t}$), and a forget gate ($f_{t}$) in addition to the hidden state ($h_{t}$) in traditional recurrent neural network (RNN).

The LSTM structure can be considered an extended version of RNNs [20]. The LSTM networks utilizes long and short-term memory to keep track of signal variations. As shown in Figure 1, each basic LSTM cell is equipped with three gates: An input gate, an output gate, and a forget gate.

Mathematically, the LSTM structure can be formulated as follows:

Forget Gate:

(1) $$f_{t}=\sigma_{g}\left(W_{f}x_{t}+U_{f}h_{t-1}+b_{f}\right)$$

Input Gate:

(2) $$i_{t}=\sigma_{g}\left(W_{i}x_{t}+U_{i}h_{t-1}+b_{i}\right)$$

Cell state update:

(3) $$C_{t}=f_{t}⊙C_{t-1}+i_{t}⊙\sigma_{c}\left(W_{c}x_{t}+U_{c}h_{t-1}+b_{c}\right)$$

Output Gate:

(4) $$O_{t}=\sigma_{g}\left(W_{o}x_{t}+U_{o}h_{t-1}+b_{o}\right)$$

Output:

(5) $$h_{t}=O_{t}⊙\sigma_{h}\left(C_{t}\right)$$

where $h_{t-1}$ and $C_{t-1}$ are the output and state of the previous LSTM cell respectively. $x_{t}$ is the input vector of the LSTM unit. $W_{*}$,$U_{*}$, and $b_{*}$ are respectively the input weight matrix, the recurrent weight matrix, and the bias term for the gate denoted by *∈{i,f,g,c}. These parameters are learned during the network training process. $\sigma_{g}$ is the sigmoid activation function while $\sigma_{c}$, and $\sigma_{h}$ are tangent hyperbolic activation functions. In the above equations, the operator ⊙ denotes the Hadamard product. The LSTM cell can update the weights according to the previous state ($C_{t-1}$) and the input gate ($i_{t}$). The capability of measuring the long interval dependency of the input signal is due to the gating mechanism which is the main characteristic of the LSTM cell [64].

#### 3.3.2. Bidirectional LSTM (BiLSTM)

In this work, we analyze the respiration recordings retrospectively and since the past, present, and future information of the time series is available at analysis time, we can use a bidirectional LSTM (BiLSTM) variant. A BiLSTM layer learns bidirectional long-term dependencies between time steps of time series or sequence data. Each BiLSTM layer consists of two layers of LSTMs: Causal and anti-causal counterparts. The anticausal LSTM which processes the time series backward in time is similar to the forward LSTM with reverse time order which leads to similar equations to the ones listed in Equations (1)–(5) but with different weights and biases $W_{*}^{{}^{\prime }}$, $U_{*}^{{}^{\prime }}$, and $b_{*}^{{}^{\prime }}$. Moreover, $h_{t-1}$ and $C_{t-1}$ are replaced respectively by $h_{t+1}^{{}^{\prime }}$ and $C_{t+1}^{{}^{\prime }}$. The outputs of the two LSTMs are then concatenated to capture the contextual information of the whole time series.

### 3.4. Network Architecture and Detection Scenarios

Most of the studies introduced in the literature proposed a feature engineering-based solution, which is highly dependent on the experts’ experience and their prior knowledge about physiological signals. In this study, to tackle the limitation of feature engineering, to learn the most prominent features, and also to increase the classification accuracy, an end-to-end deep learning technique is proposed to automatically extract features and detect apneic events in respiration time series.

**Figure 2:**
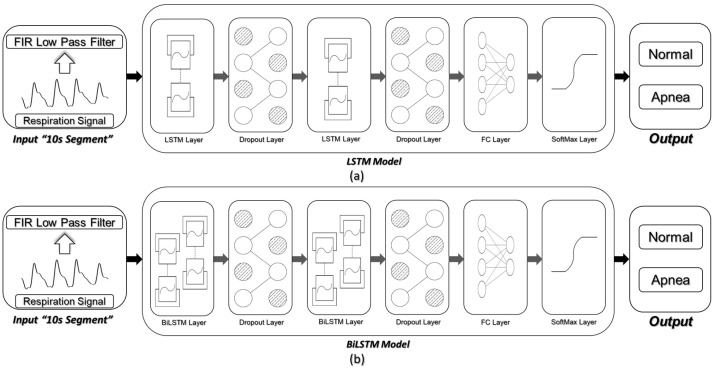
Network architecture and apnea detection scenarios. (**a**) LSTM-based approach for apnea detection. (**b**) BiLSTM-based approach for apnea detection.

As shown in Figure 2, we considered two scenarios in the proposed framework. In the first scenario, two layers of LSTMs were considered, followed by a fully connected layer (FC) and a softmax layer. In the second scenario, we replaced the LSTM layers with BiLSTM ones to evaluate the effect of using BiLSTMs in the apnea detection process as compared to standard LSTMs. Each of these modeling scenarios was evaluated on each of the three respiration signals considered in this study.

A drastic problem in most of the deep structures is overfitting. To avoid this problem, the dropout layers were used after each LSTM/BiLSTM layer. The dropout layers provide a regularization technique for deep neural networks. Using dropout technique, some of the network weights were randomly dropped during the training phase to prevent the deep neural network from overfitting [65,66].

We have scrutinized and evaluated several different combinations, to empirically identify the best architecture. To name a few, we have examined different numbers of LSTM/ BiLSTM layers, different numbers of memory cells per layer, and different numbers of fully connected layers.

### 3.5. Evaluation of Detection Results

#### 3.5.1. Classification Performance over Detection Windows

Since the proposed framework detects apnea events over 10 s windows, we used a window-based approach for evaluating the detection performance of the proposed algorithms. For each of the respiration signals considered in this study, a decision is obtained for all 10 s windows within the testing data set (Det.). This decision is then compared to the manual scoring for the corresponding windows (Ref.). Each window is then labeled as a true positive (TP), true negative (TN), false positive (FP) or false negative (FN) as illustrated in the binary classification function shown in Table 2. Det. =+1 denotes a detection of apneic window and Det. =0 denotes a detection of normal respiration window and the same analogy applies to Ref. =+1 and 0 with respect to the manual apnea annotations.

The sum of the number of windows in each group will determine the window-based classification metrics. Due to the class imbalance problem (Table 1), the classical way of considering only accuracy (ACC) as a performance metric would not allow one to fully characterize the ability of the proposed framework to detect the apneic events in respiration time series. Therefore, true positive rate (TPR), true negative rate (TNR), positive predictive value (PPV), and negative predictive value (NPV) will be used in addition to ACC as statistical measures to evaluate the performance of the proposed framework. Moreover, to account for the TPR/PPV tradeoff, the $F_{1}$ score will be reported to provide a comprehensive idea on the overall performance by considering TP and FP detections simultaneously. Mathematically, this can be expressed as follows:

(6) $$\begin{array}{ccc} TPR & = & \frac{\sum TP}{\sum TP+\sum FN}\times 100\% \end{array}$$

(7) $$\begin{array}{ccc} TNR & = & \frac{\sum TN}{\sum FP+\sum TN}\times 100\% \end{array}$$

(8) $$\begin{array}{ccc} PPV & = & \frac{\sum TP}{\sum TP+\sum FP}\times 100\% \end{array}$$

(9) $$\begin{array}{ccc} NPV & = & \frac{\sum TN}{\sum TN+\sum FN}\times 100\% \end{array}$$

(10) $$\begin{array}{ccc} ACC & = & \frac{\sum TP+\sum TN}{\sum TP+\sum FP+\sum FN+\sum TN}\times 100\% \end{array}$$

(11) $$\begin{array}{ccc} F_{1} & = & 2\frac{TPR.PPV}{TPR+PPV}\times 100\% \end{array}$$

#### 3.5.2. Receiver Operating Characteristics (ROC) Curve

The receiver operating characteristics (ROC) curve is a graphical tool that demonstrates the classification performance of a specific classifier as the classification threshold is varied [67]. This curve is created by plotting the TPR against the false positive rate (FPR =100%−TNR) at different classification thresholds. The area under receiver operating characteristics curve (AUC) reflects the overall ability of the classification model to detect sleep apnea events within respiration signals of patients. Furthermore, the ROC curve provides a convenient way for selecting the threshold that provides the maximum classification TPR while not exceeding a maximum allowable FPR level [68].

## 4. Results

### 4.1. Experimental Setting and Network Optimization

During training, different parameters of the networks and layers were explored using the training data set. The LSTM/ BiLSTM networks were used to extract temporal features. Experimental testing and optimization over the training data set resulted in setting the number of memory cells to 100 and 40 in the first and second LSTM layers respectively. Moreover, the number of memory cells for first and second BiLSTM layers were set to 100 (100×2 LSTMs) and 40 (40×2 LSTMs) respectively.

**Figure 3:**
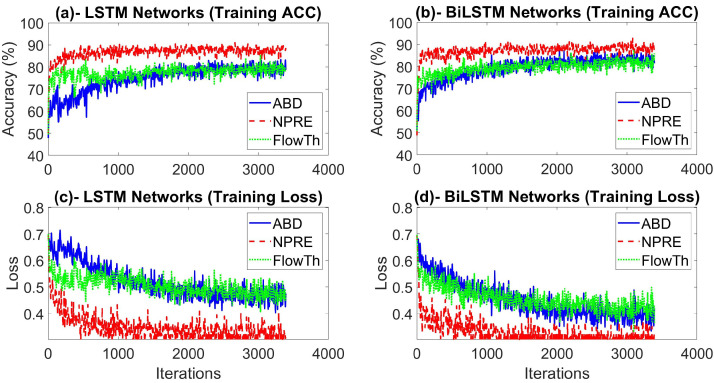
Training performance for apnea detections with different models and different respiration signals over 30 training epochs (3400 iterations). (**a**,**b**) show training accuracy for LSTM, BiLSTM networks respectively. (**c**,**d**) show training loss for LSTM, BiLSTM networks respectively.

To tackle the overfitting problem, we applied the dropout technique with the probabilities of 0.4 and 0.2 after the first and second LSTM/ BiLSTM layers respectively. This method randomly drops respectively 40% and 20% of the weights during the training phase. The Adam (adaptive moment estimation) optimizer was used as a solver which is widely used with RNNs [69]. The training process was run for 30 epochs, where an epoch equals one full cycle over the training samples. The mini batch size for gradient descent, which represents the number of training samples in each iteration to update to the weights and biases of the network, was set to 512 samples. The initial learning rate was set to 0.001 and it was updated according to a piecewise schedule that halves the learning rate every five epochs. Furthermore, the training data was shuffled at every epoch to ensure maximum representability and less variance in the learning process. The methods were all implemented on MATLAB R2020a. Figure 3 shows the accuracy and loss functions during training for the LSTM- and BiLSTM-based detection models with each of the respiration signals considered in this study. As shown in the Figure, the highest accuracy and lowest loss have been achieved with the NPRE signal detections.

### 4.2. Overall Performance over Different Respiration Signals

We first conducted an overall comparative analysis including the proposed two detection scenarios with the FlowTh, NPRE, and the ABD signals. Table 3 and Table 4 summarize the overall performance for the LSTM- and BiLSTM-based detection models respectively over 20% hold out PSG test data with respect to each of the 3 respiration channels under consideration. A total of 3 separate trials were performed for each of the respiration signals with each of the proposed detection scenarios. Table 3 and Table 4 report the best fit results for the best trial along with the standard deviation on each of the performance indices obtained from the three trials.

The ACC values are generally high for different signals and different detection scenarios. This indicates an overall high classification accuracy of the proposed framework. Although ACC is the classical metric for evaluating classification performance, it is not enough in our problem due to the high class imbalance between apnea and normal respiration segments, which is a typical challenge for detecting sleep breathing disorders. It can also be noticed that both modeling schemes achieved generally high TNR values indicating that the proposed framework could successfully identify regions of normal respiration. Moreover, the very high NPV in both detection scenarios reflect the robustness of the proposed framework in detecting normal respiration, regardless of the respiration signal considered.

**Figure 4:**
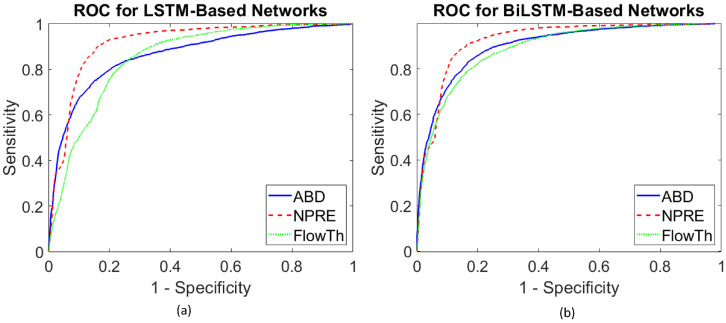
Receiver operating characteristics (ROC) curves for apnea detections with different respiration signals. (**a**) LSTM-based approach for apnea detection. (**b**) BiLSTM-based approach for apnea detection.

As shown in the tables, both detection schemes showed high AUC values over the three respiration signals indicating an excellent ability of the proposed framework in detecting sleep apnea events. This can also be verified by looking at Figure 4, which plots the ROC curves for the proposed detection models with each of the respiration signals.

For the LSTM-based detection model, Table 3 shows that the NPRE signal achieved the highest performance in detecting sleep apnea as reflected by all the binary classification metrics considered in this study compared to the FlowTh and ABD respiration signals. This was statistically validated using Friedman’s test (*p*-value = 0.009). Most importantly, TPR values reflect an excellent ability to detect apneic events using NPRE while maintaining a PPV rate close to those obtained with the other respiration signals. The $F_{1}$ score for the LSTM-based detection is significantly larger with the NPRE signal than the other two signals confirming that the overall classification performance with the NPRE is superior to the other two signals.

LSTM-based detection with the FlowTh signal achieved a relatively high TPR accompanied with low TNR results. On the other hand, the LSTM-based detection model with the ABD signal achieved low TPR along with high TNR and relatively higher PPV than the LSTM-based detection with the FlowTh signal, resulting in an overall higher $F_{1}$ score for the ABD signal compared to the FlowTh.

Table 4 indicates that the using the BiLSTM-based detection model improved the classification performance significantly with the ABD signal (*t*-test, *p*-value = 0.009) and less significantly with FlowTh signals (*t*-test, *p*-value = 0.160). The overall performance with the ABD signal is still better than the one with the FlowTh signal as reflected by the $F_{1}$ score and AUC values achieved with these signals. Interestingly, the NPRE signal still achieves the highest classification performance with the BiLSTM-based model among other signals using the same network (Friedman’s test, *p*-value = 0.069). No significant change in the detection performance with the NPRE signal is achieved by going from the LSTM-based model to the BiLSTM-based model (*t*-test, *p*-value = 0.353).

### 4.3. Individualized Patient Based Performance for the Best Detection Scenarios

Our results clearly illustrate that the NPRE signal provided the best apnea detection results with the two proposed models achieving the best performance over all the metrics considered in this study. To comprehensively evaluate the proposed modeling schemes over individual patients, we employed an LOO testing approach. In this method, we hold out one patient PSG data, build the model using the remaining PSG data, and the model is then evaluated on the held out patient data. This process is repeated over all patients until we test them all. This test approach was applied on each of the proposed detection scenarios with the NPRE signal since this signal achieved the best performance results.

Table 5 and Table 6 summarize respectively the performance of the LSTM- and BiLSTM-based detection models in detecting sleep apnea events over individual test patients using the NPRE signal. As shown in these tables, both detection models show excellent apnea detection results over individual patients. The BiLSTM-based detection model showed (statistically) non-significant improvement in detection results compared to the LSTM model over individual patients (*t*-test, *p*-value = 0.523). Nevertheless, both detection models provide promising results achieving relatively high performance measures with respect to the metrics considered in this study.

## 5. Discussion

The study proposed a novel method for automatic detection of apneic events based on deep RNN from a single channel respiration signal. Two RNN detection schemes were employed. The first model uses an LSTM-based detection network while the second model uses a BiLSTM-based detection network. Three respiration signals were considered and tested separately with the proposed framework. These signals are the oronasal airflow signal (FlowTh), the nasal pressure signal (NPRE), and the Abdominal RIP Signal (ABD).

Although both signals detect the respiratory activity during PSG, the signal from the oronasal thermal airflow has different characteristics than the one recorded by the nasal pressure transducer. The oronasal thermal airflow signal is not proportional to flow and typically overestimates flow as flow rates decrease, making it more sensitive for detecting (significant) flow limitations that occur during different types of apnea events [70,71]. On the other hand, the nasal pressure sensor is less sensitive to low levels of flow and it is also not capable of detecting oral airflow [28,29]. Although the pressure signal can be used to provide an estimate of airflow by applying a square root transformation, this affects the accuracy of the transformed signal making it easily susceptible to noise and deteriorates over night time [72]. To overcome weaknesses in both sensors, AASM recommends the use of these two sensors in PSG diagnostic studies for sleep breathing disorders [25].

The thoracic (THO) and abdominal (ABD) movement signals, captured using wearable bands/belts, are recommended by AASM as an alternative source for detecting sleep apnea/hypopnea events [25]. The potential advantage of the ABD/THO signals over nasal signals that they provide indirect access to respiration airflow and that they do not depend on the patient having to breathe solely through the nose [73].

In the recent years, several studies have focused on automated detection of sleep apnea events based exclusively on the analysis of a single respiratory signal. Many studies used the thermal oronasal airflow sensors to build classical machine learning methods [34,38,39,40,41,44,74,75,76] while others used the nasal pressure signal [11,17,35,43,45,77,78,79,80,81]. Although being much less widely explored, the use of respiratory wearable belts in automated detection of sleep apnea also showed very good results [73,82,83,84,85]. These signals fundamentally vary with the sensing mechanisms that record them. Additionally, these signals are highly dependent on many factors, such as a calibration of the measuring device, physiological conditions of the patient, and presence of artifacts [86]. These factors limited the clinical adoption of respiratory signals for automated apnea detection as well as the ability of the proposed methods to generalize over different device/experimental setups and patient populations [68]. Furthermore, the differences among these signals further complicate calculations and computations to extract handcrafted feature sets prior to processing them with machine learning algorithms. Consequently, very limited success was reported on the validation of respiratory-based apnea detection algorithms using features optimized from different types of respiratory sensors [35,85].

Our study advances the state of art by developing a unified end-to-end RNN-based deep learning framework for automatically extracting temporal features and detecting sleep apnea events from single channel respiration signals. The proposed framework is distinct from many existing methods (SVM, LDA, etc.) through eliminating the need for extracting a set of human-engineered features in order to detect apnea events with classical classification models. Not only will the proposed framework eliminate the step of manual extraction for the feature set, but it will also provide more robust and optimized automatically extracted features leading to more consistent performance in apnea detection. Most importantly, the framework is flexible to work with different PSG signals as it only needs a noise-filtered respiration signal as an input. This will potentially allow the presented framework to easily generalize over broader experimental settings and various respiratory sensors.

A recent comprehensive survey showed that the vast majority of deep learning methods for sleep breathing disorders have been devoted to ECG signals [46]. Few studies considered single channel respiration inputs for deep learning apnea detection models [58,59,60,61]. Convolutional neural networks were used in [58,59,61] while [60] used human-engineered features as an input to a deeply stacked feed-forward neural network but none of them have evaluated recurrent neural networks. Although CNNs are widely used in deep learning methods, they require very high computational power as opposed to RNNs and are also designed to work with images unlike RNNs that are fundamentally used for signals with temporal dependencies. The work of [58,59,60] considered only the FlowTh signal but did not consider NPRE and ABD signals. The work of [61] considered only the NPRE signal and did not evaluate other respiration signals. Similarly, the work of [57] included ECG and ABD/THO signals but ignored primary respiratory flow signals NPRE and FlowTH. Future work may consider a comprehensive comparison between CNN-based and RNN-based methods as well as hybrid methods that combine both types of networks over larger data sets and wider subsets of signals.

Our results show that the best detection results were obtained with a nasal pressure signal compared to oronasal airflow and the abdominal respiratory inductance plethysmography. NPRE signal maintained the highest apnea detection results with the two models analyzed. There was not a significant difference in the detection performance between LSTM- and BiLSTM-based models when using the NPRE signal for apnea detection. Our results with the proposed deep learning framework agree with previous studies that compare NPRE with other respiration signals. In particular, ref [78] compared respiratory flow signals using FlowTh and NPRE for patients with obstructive sleep breathing disorders. Results of this study indicate that measuring airflow with an NPRE device is superior to measuring airflow using FlowTh technology. The study demonstrated that FlowTh measurements significantly underestimated both apneic and hypopneic events and that measuring the flow signal using NPRE during sleep studies was simple and more accurate than FlowTh. Furthermore, [87] found that almost all events detected by a FlowTH were also detected using NPRE, but that events completely missed by a FlowTh were recognized in NPRE measurements. Finally, [29,88] reported increased TPR for NPRE measurements compared to FlowTH measurements and compared to RIP movement measurements [89].

There are some limitations in our study. We did not consider the hypopnoea events because of their rarity in our data set which did not allow characterizing them separately. The proposed deep RNN framework is unaware of the starting and ending point of apnea events because of performing event-based detection that can only detect the presence or absence of apnea events. To test the algorithm in a practical setting, we did not remove the noise events like snoring and movement artifacts. We used only basic memory cells of LSTM and BiLSTM, and did not use any variation of LSTM/BiLSTM or gated recurrent units (GRU). Finally, a small number of subjects were used as a proof of concept for the proposed method. Future work will focus on resolving these limitations and thereby facilitating the development of more robust deep learning models from respiratory signals.

## 6. Conclusions

In this study, we demonstrated the use of deep RNN models in automatic detection of apneic events using a single channel respiratory signal. Two major RNN models were utilized: LSTM and BiLSTM. Furthermore, the proposed framework was evaluated on 3 different respiration signals including the oronasal thermal airflow sensor, the nasal pressure sensor, and the abdominal respiratory inductance plethysmography sensor. The best detection results were obtained with the nasal pressure signals in both detection models. The BiLSTM-based model improved the performance with the oronasal thermal airflow signals and the abdominal respiratory inductance plethysmography signal compared to the LSTM-based one. The BiLSTM model with the nasal pressure signal achieved an overall event-based test performance of TPR=90.3%, TNR=83.7%, and AUC=92.4% in apnea detections. The proposed framework was further validated on a patient level achieving a mean performance of TPR = 86.0%, TPR = 84.1%, and AUC = 92.3% with a BiLSTM model tested over the NPRE signal of individualized patients. Our results provide insights for the effectiveness of the proposed RNN model in diagnosing and screening sleep apnea patients, which can be highly valuable for standard PSG systems.

## Figures and Tables

**Table 1:** Segments of training and testing data sets. The distribution of data entails randomly dividing each label to 80% for training and 20% for testing.

Data Summary			
Labels/Segments	Training Set	Testing Set	Total
Normal	29,078	7270	36,348
Apnea	7493	1873	9366
Total	36,571	9143	45,714

**Table 2:** Classification performance over detection windows.

	Ref = +1	Ref = 0
Det. = +1	TP	FP
Det. = 0	FN	TN

**Table 3:** Overall test performance over 20% hold dut data for LSTM-based detection model. The nasal pressure (NPRE) signal shows the highest classification performance with the LSTM-based detection model.

LSTM-Based Network—Overall Test Performance over 20% Hold Out of Data							
**Input Signal**	TPR%	TNR%	ACC%	PPV%	NPV%	AUC%	$F_{1}\%$
NPRE	90.0 (0.6)	83.8 (0.5)	85.1 (0.3)	58.9 (0.6)	97.0 (0.2)	91.7 (0.2)	71.2 (0.3)
ABD	77.0 (1.1)	82.7 (1.5)	81.5 (1.1)	53.4 (2.0)	93.3 (0.3)	86.5 (0.8)	63.1 (1.5)
FlowTh	85.1 (1.7)	72.9 (2.1)	75.4 (1.4)	44.7 (1.4)	95.0 (0.4)	85.1 (1.8)	58.6 (0.9)

**Table 4:** Overall test performance over 20% hold out data for BiLSTM-based detection model. Replacing LSTM layers with BiLSTM ones improved the overall classification capability for ABD and FlowTh signals but the NPRE signal still shows the highest classification performance with the BiLSTM-based architecture.

BiLSTM-Based Network: Overall Performance over 20% Hold Out of Data							
**Input Signal**	TPR%	TNR%	ACC%	PPV%	NPV%	AUC%	$F_{1}\%$
NPRE	90.3 (0.5)	83.7 (0.6)	85.0 (0.4)	58.8 (0.9)	97.1 (0.1)	92.4 (0.3)	71.2 (0.5)
ABD	78.5 (2.7)	85.9 (0.7)	84.4 (1.1)	59.0 (2.0)	94.0 (0.8)	90.1 (2.1)	67.4 (2.3)
FlowTh	80.5 (1.7)	81.6 (2.3)	81.4 (1.5)	53.0 (2.3)	94.2 (0.3)	89.0 (0.3)	63.9 (1.3)

**Table 5:** Individualized patient test performance using the LSTM-based detection scheme and the NPRE Signal.

Leave One Out Test Results—LSTM-Based Detection Model with NPRE Signal								
**#**	**Patient ID**	TPR%	TNR%	ACC%	PPV%	NPV%	AUC%	$F_{1}\%$
1	1	90.7	88.0	89.1	83.8	93.2	93.4	87.1
2	2	66.9	93.8	91.0	55.6	96.1	89.6	60.7
3	3	97.9	74.7	80.9	58.3	99.0	91.8	73.1
4	4	94.8	92.1	92.6	75.7	98.5	96.6	84.2
5	8	81.3	89.4	87.5	70.6	93.8	91.5	75.6
6	9	92.6	86.1	88.4	78.2	95.6	94.6	84.8
7	10	41.9	98.9	98.0	37.5	99.1	93.9	39.6
8	15	91.7	77.0	78.8	35.3	98.5	90.9	51.0
9	16	89.8	76.7	77.1	12.9	99.5	91.4	22.6
10	17	97.4	78.2	84.0	65.7	98.6	92.7	78.5
11	18	97.0	50.6	59.5	31.9	98.6	74.8	48.0
14	21	96.3	70.9	82.8	74.3	95.7	89.7	83.9
15	22	77.3	94.5	93.1	56.4	97.9	93.4	65.2
16	23	93.9	92.9	93.8	62.0	99.2	97.6	74.7
17	24	88.7	76.3	81.9	75.4	89.2	90.1	81.5
	Average	86.7	83.0	85.4	57.4	96.9	91.7	69.0

**Table 6:** Individualized patient test performance using the BiLSTM-based detection scheme and the NPRE signal.

Leave One Out Test Results—BiLSTM-Based Detection Model with NPRE Signal								
**#**	**Patient ID**	TPR%	TNR%	ACC%	PPV%	NPV%	AUC%	$F_{1}\%$
1	1	91.7	89.5	90.4	85.7	94.0	95.8	88.6
2	2	63.3	94.5	91.2	56.9	95.7	88.7	59.9
3	3	97.9	72.6	79.3	56.3	99.0	92.0	71.5
4	4	97.1	87.4	89.4	66.7	99.1	97.0	79.1
5	8	74.8	92.0	87.9	74.5	92.1	92.4	74.6
6	9	94.7	84.3	87.9	76.5	96.7	95.2	84.6
7	10	62.8	98.3	97.7	37.0	99.4	96.9	46.6
8	15	92.0	78.7	80.3	37.2	98.6	91.6	53.0
9	16	88.0	80.1	80.4	14.6	99.4	92.2	25.0
10	17	96.5	81.0	85.6	68.5	98.2	95.9	80.1
11	18	96.6	51.9	60.5	32.4	98.5	73.2	48.5
12	19	78.0	95.9	94.6	58.7	98.3	96.7	67.0
13	20	90.2	74.7	77.6	44.8	97.1	89.8	59.9
14	21	92.9	75.6	83.7	76.8	92.5	90.9	84.1
15	22	72.5	94.5	92.7	54.8	97.4	92.4	62.4
16	23	93.5	93.3	93.3	60.2	99.3	97.7	73.2
17	24	79.0	85.6	82.6	81.7	83.2	90.0	80.3
	Average	86.0	84.1	85.6	57.8	96.4	92.3	69.2
